# Perceptions and Beliefs About Preconceptional Care Among Primary Healthcare Workers in Jeddah City, Saudi Arabia: An Analytical Cross-Sectional Study

**DOI:** 10.7759/cureus.41178

**Published:** 2023-06-30

**Authors:** Nada Fussi, Najlaa Mandoura

**Affiliations:** 1 Preventive Medicine, Ministry of Health, Riyadh/Jeddah, SAU; 2 Epidemiology and Public Health, Directorate of Health Affairs, Public Health Division, Jeddah, SAU

**Keywords:** primary healthcare workers, preventive services, primary care, pregnancy, preconception care

## Abstract

Introduction

Preconception care (PCC) is one of the important aspects of reproductive health and family planning, from the preventive aspect as primordial prevention for future offspring and primary prevention for females before pregnancy. However, there is no written protocol about PCC and it is not routinely practiced in Saudi Arabia. This study aimed to assess the perceptions and beliefs among care workers regarding PCC.

Methods

A cross-sectional study was conducted on general practitioners (GP), family physicians (FP), practitioner nurses (PN), and midwives (MW) in primary healthcare centers (PHC) in Jeddah City using a validated questionnaire that assesses their preconception practices, perceptions, and beliefs.

Results

This study included 201 participants, of whom 98.5% were Saudi nationals and 80.1% were female. Most (64.7%) were 30-39 years old, followed by 40-49 years old (21.9%). The majority (67.7%) were married and had one or two children (37.3%). Most (36%) were practitioner nurses, followed by family physicians (31%), and had 11-15 years of experience (32%), followed by six to 10 years of experience. The majority (44%) reported providing PCC one to five times last month. Of all participants, 72.63% agreed that PCC affected pregnancy outcomes, and 83% agreed that PCC is important. However, 51.7% agreed there is not enough time to provide PCC services. The service rated as the highest priority was providing advice regarding smoking cessation (82.1%), alcohol cessation (84.6%), control of chronic diseases (85.1%), and information about drug use (86.6%). Most participants rated rubella screening as highly important (89.9%), followed by hepatitis screening (88.6%). Family physicians and practitioner nurses perceived PCC as more important than general practitioners and midwives (p=0.026) and were more likely to perceive hospitals as the optimal setting for PCC (p=0.015). General practitioners were more likely to believe in the insufficient evidence base for PCC (p < 0.001).

Conclusion

The study found that healthcare workers had good perceptions, knowledge, and attitudes toward the PCC, but their practice was poor. Most lacked formal training and had differing perspectives on PCC, depending on their professions. The findings could inform strategies and measures to improve PCC practice among healthcare workers and raise awareness as well as capacity building by enhancing the training of healthcare workers.

## Introduction

Preconception care (PCC) is a set of interventions to identify and modify biomedical, behavioral, and social risks to a woman's health or pregnancy outcome through prevention and management [1,2]. Those interventions should be made before or early in pregnancy to have maximal impact. PCC is defined by the World Health Organization (WHO) as “the provision of biomedical, behavioral and social health interventions to women and couples before conception occurs, aimed at improving their health status and reducing behaviors and individual and environmental factors that could contribute to poor maternal and child health outcomes” [3]. Risk assessment during PCC includes screening, personal and family history, physical exam, laboratory screening, preventive health, reproductive plan, nutrition supplements, weight, exercise, vaccinations, injury prevention, exposures to medications (teratogens) due to chronic diseases, substance abuse, environmental hazards, and toxins [1,4,5]. PCC focuses on helping individuals via services, interventions, support, and advice to plan and be fit for pregnancy and minimize risk factors for poor birth outcomes [6]. PCC is advantageous for all couples wishing to become parents and is advised for those with reproductive problems [7]. It is a chance to improve health, spot and control any hazards, and raise the likelihood of a good pregnancy and healthy child.

In Saudi Arabia, Sabr et al. found that Saudi women had moderate knowledge and good perceptions of PCC [8]. One of the most recognized preconception interventions is the consumption of folic acid, which prevents congenital neural tube defects [9]. In Yanbu City, Saudi Arabia, it was reported that less than half of the women consumed folic acid daily, and only 46.2% started consuming it during the first month of pregnancy [10]. Moreover, obesity (BMI ≥ 30), which is a risk factor for poor birth outcomes, is estimated to be 24.7%, more in females than males in Saudi Arabia [11]. Research has shown that babies born to obese women have a higher risk of fetal death, stillbirth, congenital abnormality, shoulder dystocia, macrosomia, and subsequent obesity [12]. Apart from obesity, Saudi women are also at high risk of other lifestyle-related morbidities due to recent socioeconomic changes the Kingdom of Saudi Arabia has undergone [13]. This highlights the need for enhancing PCC to minimize risk factors for poor pregnancy outcomes and optimize maternal and child health. Knowing the perceptions of primary care workers toward PCC would help establish PCC into existing healthcare services in Saudi Arabia and raise awareness of PCC among primary healthcare workers. Therefore, this study assessed the perceptions and beliefs about the PCC and associated factors among healthcare workers in Jeddah city.

## Materials and methods

Study design

An analytical cross-sectional study was conducted in Jeddah City in March 2023. The city has around 4.08 million, and it is the second largest city after Riyadh. Jeddah City comprises 49 primary care centers distributed among five general hospitals. We used an analytical cross-sectional, quantitative, non-experimental research design that gathers data from a group of subjects at only one point in time to measure the association between an exposure and a disease, condition, or outcome within a defined population, often utilizing questionnaires [14,15].

Study population

We included all general practitioners (GP), family physicians (FP), practitioner nurses (PN), and midwives (MW), with more than six months of experience and three months working in the PHC and caring for pregnant women. We excluded the administration workers, dentists, and healthcare workers with a work experience of less than six months or less than three months working in the PHC or those who worked outside Jeddah city.

Sample size

Using the Raosoft® software (Raosoft Inc, Seattle, WA), we calculated the minimum sample size of 202, considering an acceptable margin of error = 0.05 and a response rate estimated to be 80%. According to the Directorate of Health Affairs, there are about 50 PHCs in Jeddah City, consisting of 1500 healthcare workers, among whom 1100 fulfilled our inclusion criteria.

Sampling technique

Multi-stage sampling technique was used to select eligible participants. First stage: Primary healthcare centers (PHCs) were stratified into 5 strata according to five hospitals in Jeddah city that supervise them. Second stage: PHCs were randomly selected to form 34 clusters Third stage: 7 to 8 healthcare workers were selected from each PHC to make up 202 total participants.

Data collection tool

A validated questionnaire from a similar published study by Heyes et al. was used for data collection [16]. The questionnaire was devised and refined in discussion with GPs and primary care researchers at the Institute of General Practice at Sheffield University, England. Then the first author (Fussi, N) got approval from the researcher of the previous similar research via E-mail contact. The questionnaire was pilot tested on 20 healthcare providers for its reliability and wording. The Cronbach Coefficient Alpha test was done, and the questionnaire scored 87%. The pilot study results were used for improving word clarity and were not included in the main study.

The questionnaire collects sociodemographic questions such as age, gender, nationality, marital status, married years, number of children, geographical region in Jeddah, current occupational position, and years of experience in the medical field. It then assesses PCC practices and perceptions toward PCC.

Variable measurements

Age was categorized with 10-year intervals, starting from 20 years up to 59 years, gender was binary (male or female), and nationality was also binary (Saudi or non-Saudi). Marital status was categorized into: “Married,” “Single,” “Divorced,” and “Widow.” The number of children was categorized into: “1-2 children,” 3-5 “children,” “over 5 children,” and “No child”. The geographical region was categorized into North, South, East, West, and Middle Jeddah. Years of experience were categorized into: “1-5 years”, “6-10 years”, “11-15 years”, and “Above 15 years”. Times of PCC provision were categorized into: “Nil,” “1-5 times”, “6-10 times”, and “above 10 times.”

The questionnaire measures practices, beliefs, and perceptions using questions regarding the PCC service package, the main features of PCC, perception of PCC in themes of personal qualification, priority, the pattern of provision, belief in efficacy, training received, and perceived barriers to providing PCC. PCC practices, beliefs, and perceptions were measured using Likert scales (from 1 = strongly disagree to 5 = strongly agree, and 5 = highest importance to 1 = No importance, respectively). Then, the perception scores are calculated, with a minimum score of 14 and a maximum of 55.

Data collection technique

Anonymous questionnaires were distributed via an online link (Google Forms) to participants with an invitation letter and a consent form. The participants were asked to sign their consent forms before responding to the questionnaires.

Statistical analysis

The data were analyzed using Statistical Package for Social Sciences (SPSS) version 21 (IBM Corp., Armonk, NY). A confidence level of 95% (95% CI) was adopted throughout the study, and a P value less than 0.05 was considered a significance level. Continuous variables (perceptions) were presented as mean and standard deviation (SD) while categorical variables (socio-demographics) were presented as frequency distribution (n) and percentages (%). The chi-square test was used to assess the association between the outcomes (perception and beliefs scores) and the independent variables (socio-demographics).

Ethical considerations

Ethical approval from the research committee of the Saudi Board of Preventive Medicine (SBPM) and the Institutional Review Board (IRB) was obtained before starting data collection (ethical approval number: A01593). Participation was voluntary, and participants were allowed to withdraw at any time.

## Results

We included 202 participants, and one withdrew later. Among the 201 remaining, 98.5% were Saudi nationals, 80.1% were female, and 19.9% were male. Of all participants, 64.7% were 30-39 years old, followed by those aged 40-49 (21.9%). The majority (67.7%) were married, 22.4% were single, and 9.5% were divorcees. Most participants had one or two children (37.3%). Table 1 provides further details of the demographic data of the participants.

**Table 1:** Demographic data of the participants

Variable	Frequency	Percentages
Gender		
Male	40	19.9
Female	161	80.1
Age group		
20-29 years	15	7.5
30-39 years	130	64.7
40-49 years	44	21.9
50-59 years	12	6.0
Nationality		
Saudi	198	98.5
Non-Saudi	3	1.5
Marital Status		
Divorced	19	9.5
Married	136	67.7
Single	45	22.4
Widower/Widow	1	0.5
Number of children		
1 – 2 children	75	37.3
3 – 5 children	61	30.3
>5 children	14	7.0
No children	51	25.4
Geographical Region		
East Jeddah	42	20.9
Middle Jeddah	29	14.4
North Jeddah	90	44.8
South Jeddah	28	13.9
West Jeddah	12	6.0

**Figure 1:**
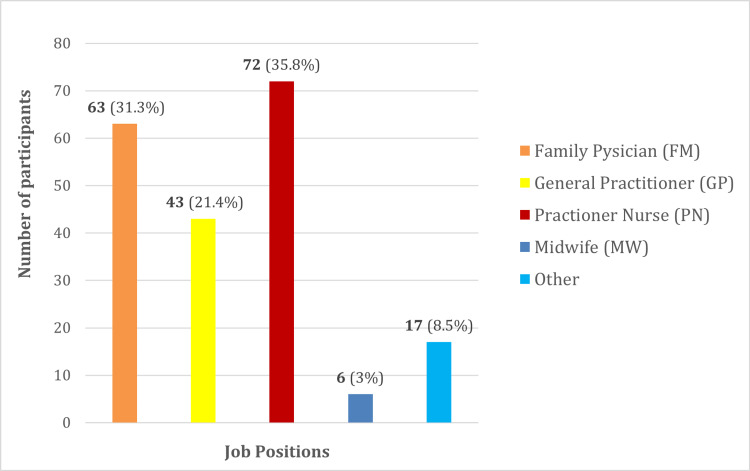
Job positions of the participants (n=201)

The majority were practitioner nurses (n=72), followed by family physicians (n=63) and general practitioners (n=43) (Figure 1).

**Figure 2:**
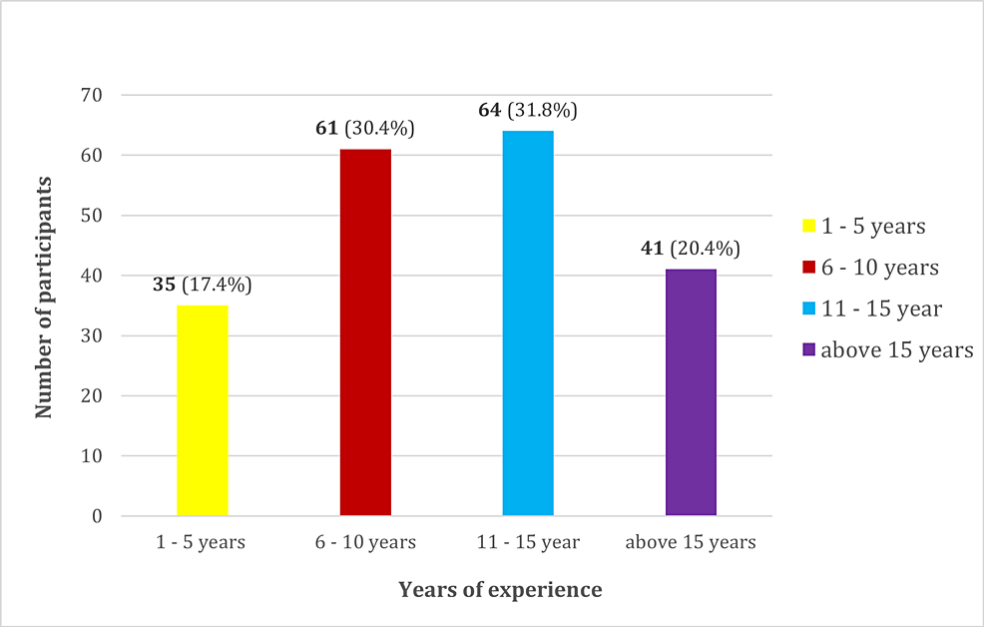
Years of experience of the participants

Regarding years of experience, the majority of participants (n=64) had 11-15 years of experience, followed by 6-10 years of experience (n=61) (Figure 2).

**Figure 3:**
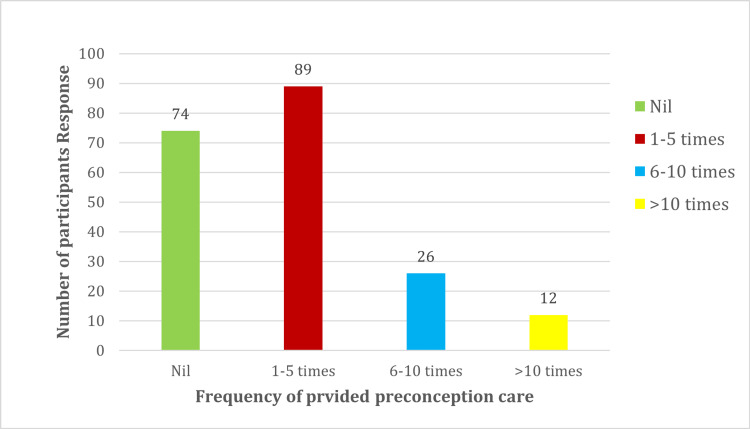
Times preconception care was provided by the participants

The majority of participants (n=89) reported having provided PCC one to five times in the last month, followed by 26 of those who had provided it 6-10 times (Figure 3).

Table 2 displays the perceptions of healthcare workers regarding PCC. The majority (72.63%) disagreed with the statement that PCC has no effect on pregnancy outcomes. On the other hand, 83% agreed that PCC is important for women of childbearing age. Nearly half of the participants (47.3%) disagreed that PCC is a luxury service while only 19% expressed uncertainty. About 40.8% agreed that hospitals are the best place for PCC. However, 42.3% agreed that population planning for pregnancy often does not occur. A similar proportion (42.8%) agreed that PCC is a high priority in their workload.

**Table 2:** Perception of healthcare workers regarding preconception care (n=201)

Perceptions	Strongly Disagree	Disagree	Uncertain	Agree	Strongly Agree
1. Preconception care does not have any effect on pregnancy outcome	113(56.2)	33(16.4)	8(4.0)	26(12.9)	21(10.4)
2. Preconception care is an important health issue for women of childbearing age	20(10)	6(3)	8(4)	62(30.8)	105(52.2)
3. A dedicated clinic for preconception care is a luxury service	50(24.9)	45(22.4)	39(19.4)	34(16.9)	33(16.4)
4. A hospital setting is the best place to provide preconception care	32(15.9)	46(22.9)	41(20.4)	53(26.4)	29(14.4)
5. In this practice population planning for a pregnancy often does not happen	13(6.5)	30(14.9)	73(36.3)	60(29.9)	25(12.4)
6. Preconception care is a high priority in my workload	11(5.5)	39(19.4)	65(32.3)	60(29.9)	26(12.9)
7. There is not enough time to provide a preconception clinic	23(11.4)	37(18.4)	37(18.4)	69(34.3)	35(17.4)
8. As a practitioner I do not have the appropriate skills to offer preconception care	39(19.4)	65(32.3)	40(19.9)	44(21.9)	39(19.4)
9. There is little evidence base for preconception care	30(14.9)	51(25.4)	54(26.9)	48(23.9)	18(9.0)
10. General practice is the best place to provide preconception care	10(5.0)	19(9.5)	44(21.9)	85(42.3)	43(21.4)

Slightly over half (51.7%) agreed that there is not enough time to provide a preconception clinic while the same percentage disagreed that, as practitioners, they lack the appropriate skills to offer PCC. Approximately 40.3% disagreed that PCC has little evidence base, and almost two-thirds (63.7%) agreed that general practice is the optimal setting to provide PCC.

When asked to rate various aspects of PCC based on their importance, The highest ratings were given to advise regarding smoking cessation (82.1%), alcohol cessation (84.6%), control of chronic diseases (85.1%), and information about drug use (86.6%). A majority of the respondents considered the following topics as very important: maternity care (79.1%), advising about folic acid supplementation (74.1%), and genetic counseling (71.1%) (Table 3).

**Table 3:** The importance of different aspects of preconception care (1 = not important, 5 = very important)

	Services for preconception	5	4	3	2	1
1	Smoking advice	165(82.1)	23(11.4)	9(4.5)	1(0.5)	3(1.5)
2	Advice on alcohol use	170(84.6)	10(5.0)	10(5.0)	9(4.5)	2(1)
3	Maternity care	159(79.1)	23(11.4)	12(6.0)	2(1.0)	5(2.5)
4	Exercise advice	133(66.2)	28(13.9)	29(14.4)	5(2.5)	6(3.0)
5	Drug use advice	174(86.6)	17(8.5)	5(2.5)	4(2.0)	1(0.5)
6	Chronic diseases, for example, diabetes, epilepsy, hypertension	171(85.1)	15(7.5)	6(3.0)	8(4.0)	1(0.5)
7	Diet (including weight control)	138(68.7)	29(14.4)	22(10.9)	5(2.5)	7(3.5)
8	Folic acid advice	149(74.1)	26(12.9)	22(10.9)	3(1.5)	1(0.5)
9	Other supplements	120(59.7)	35(17.4)	34(16.9)	5(2.5)	7(3.5)
10	Food safety advice	128(63.7)	33(16.4)	24(11.9)	9(4.5)	7(3.5)
11	Occupational hazards advice	135(67.2)	33(16.4)	28(13.9)	4(2.0)	1(0.5)
12	Genetic counseling (if indicated)	143(71.1)	31(15.4)	16(8.0)	9(4.5)	2(1.0)

When it comes to screening for preconception, most participants rated rubella screening as highly important (89.9%), followed by hepatitis screening (88.6%), HIV screening (87.6%), and genital infection screening (82.6%), as shown in Table 4.

**Table 4:** Screening for preconception (1 = no important, 5 = very important)

	Screening for preconception	5	4	3	2	1
1	Rubella screening	180(89.6)	12(6.0)	3(1.5)	00	6(3.0)
2	Hepatitis screening	178(88.6)	14(7.0)	4(2.0)	5(2.5)	00
3	Nutritional status	146(72.6)	28(13.9)	25(12.4)	1(0.5)	1(0.5)
4	Cervical cytology	137(68.2)	36(17.9)	15(7.5)	5(2.5)	8(4.0)
5	HIV screening	176(87.6)	16(8.0)	7(3.5)	00	2(1.0)
6	Genital infections	166(82.6)	17(8.5)	13(6.5)	00	5(2.5)

Our study found that only 43.3% of the participants had received formal training on PCC. Among those who received training, a third (30.3%) considered it to be very useful.

During the chi-square test, agreeing responses were combined, as well as disagreeing responses. The chi-square test showed that family physicians and practitioner nurses were more inclined than general practitioners and midwives to perceive PCC as important (p=0.026) and were more likely to believe that hospitals are the optimal setting for PCC (p=0.015). In contrast, general practitioners and midwives were more likely to disagree with the statement suggesting that there is a lack of appropriate skills to provide PCC (p < 0.001). Additionally, general practitioners exhibited a higher likelihood compared to the other groups to agree that there is insufficient evidence base for PCC (p < 0.001) (Appendices).

## Discussion

Preconception care is a vital aspect of reproductive healthcare that focuses on optimizing the health of individuals before they plan to conceive [4,7]. Primary care workers, including physicians, nurses, and midwives, play a crucial role in providing this care. This study explored the perceptions and beliefs of primary care workers in Jeddah City regarding preconception care. The results could help guide measures for raising awareness regarding the importance of the PCC for improving its delivery and acceptance.

We found that over a third of participants (36.8%) had never provided preconception care, and among those who provided it, less than half had provided it one to five times and around a quarter provided it 6-10 times. This indicates the less and partial practice of preconception care compared to 87.8% reported in Kwa-Zulu-Natal, South Africa, and Nashville, USA [2,17]. A study from Ethiopia showed that most primary healthcare workers (84.7%) did not fully practice PCC [1], which aligns with our findings. Only 42.8% of participants in our study agreed that preconception care is a high priority in their workload. This poor practice is also supported by the absence of population planning for a pregnancy reported by some participants. Cultural factors and societal beliefs may influence the perception of PCC as a low priority, leading to a lack of demand and contributing to healthcare workers' limited engagement with PCC [18]. We found the mean perception score to be fairly good (33.54±6.69). Participants from Southern Jeddah and those married were more likely to score high (p<0.001, and p = 0.007, respectively). Most married couples start to plan for the families and babies they will have [19], which raises curiosity and preparations to have a good pregnancy outcome, and this can lead to acquiring a good knowledge of PCC, which is also associated with perception [4,18].

While some healthcare providers incorporate PCC into their routine practice, many focus primarily on antenatal care, with limited attention given to the preconception period [20]. This leads to missed opportunities for preventive interventions and addressing potential risk factors before pregnancy. In another study, Heyes et al. found that healthcare providers provided PCC services mainly opportunistically [16]. A Dutch study/publication indicated that almost all healthcare providers suggested the responsibility for preconception care consultations lies within primary care. Still, approximately one in seven midwives (strongly) disagreed that it is part of their job to provide preconception information to couples wishing to conceive [21]. In contrast, our study showed better attitudes because most participants agreed that preconception care is important for women of childbearing age and has a positive effect on pregnancy outcomes.

Education and training programs can play a vital role in enhancing healthcare workers' knowledge and skills in PCC. Providing targeted training on preconceptional care topics, risk assessment, and counseling techniques will empower healthcare providers to effectively incorporate PCC into their routine practice. Unfortunately, only 43.3% of the participants reported receiving formal PCC training. A study conducted in Iran showed that midwives' education influenced PCC practice [22]. In Yanbu, Saudi Arabia, Al-Marwani et al. reported that professionals other than midwives found it significantly more difficult to start a conversation about a wish to conceive compared to midwives and felt less competent in providing pre-conceptional information [10]. Maas et al. suggested a need to integrate PCC in many curricula and postgraduate courses, especially for non-midwives, to improve the delivery of PCC [21].

However, our findings showed better attitudes toward PCC importance (p=0.026) among family physicians and nurses than among midwives and general practitioners, despite general practitioners and midwives perceiving no lack of appropriate skills to provide preconception care (p < 0.001). This might be due to the high likelihood of believing that there is insufficient evidence base for preconception care (p < 0.001). These findings highlight the need for raising awareness, focusing on midwives and general practitioners about the importance of PCC. More research evidence and educational materials can help them understand the potential benefits of PCC.

Several challenges and barriers may contribute to the limited practice of PCC. Healthcare providers often have limited time during consultations to address preconceptional matters thoroughly. This results in a focus on immediate concerns and leaves little room for proactive discussions about preconception health. Our study showed that over half had not enough time to provide preconception services. Previous research identified time restrictions as one of the main limitations, in addition to the absence of women seeking PCC and many competing priorities in the general practice setting [4,23]. Despite general practice settings being too busy, almost two-thirds (63.7%) of participants in our study agreed that general practice is the optimal setting to provide preconception care. Therefore, addressing this could require expanding resources to reduce workload and integrating PCC items into the general practice guidelines.

PCC package includes discussing lifestyle modifications, including smoking cessation, alcohol avoidance, and maintaining a healthy weight, with individuals of reproductive age [24]. They may also guide the importance of folic acid supplementation, immunizations, and chronic disease management before conception [9,10]. Our participants rated smoking cessation and cessation, control of chronic diseases, and information about drug use as the highly prioritized topics to discuss during the PCC with patients. Smoking is the single most significant modifiable risk factor for poor birth outcomes, and Rashid et al. [25] showed that passive maternal smoking was associated with a decrease in birth weight and an increase in small gestational age infants in Saudi Arabia, aligning with another study by Wahabi et al. [26]. PCC is crucial for Saudi women who are at high risk of obesity, leading to significantly higher odds of gestational diabetes (OR 3.01 to 5.55), preeclampsia (OR 2.93 to 4.14), antenatal (OR 1.43) and postpartum depression (OR 1.30), and cesarean delivery (OR 2.01 to 2.26) as compared with normal-weight women [27]. Folic acid is very important for developing a healthy fetus, as it significantly reduces the risk of neural tube defects, such as spina bifida, and is recommended before conception [9,28].

Some limitations of this study include its cross-sectional incapable to determine the causality-effect relationship and being prone to selection bias. The study was conducted with an online questionnaire, which is also prone to selection bias and could lead to underreporting or overreporting, as it relies on self-reporting of participants. This study did not extensively explore the effect of PCC training on the practice among healthcare providers studied. Therefore, we recommend future broad longitudinal studies addressing the differences in perception and application of PCC among healthcare providers. Future studies should also extensively explore the effect of training, either previous or current, on perceptions and practices of PCC among healthcare providers.

## Conclusions

This study showed that healthcare workers caring for pregnant women did not practice PCC well. However, they had fairly good attitudes and knowledge of PCC and its service package. It also showed that less than half of the participants received formal training. Surprisingly, family physicians and practitioner nurses had more positive beliefs toward PCC than midwives and general practitioners, showing varying perspectives among healthcare professionals regarding PCC. This study showed misconceptions such as the lack of evidence-based current PCC guidelines and inadequate time for PCC practice among healthcare providers. The findings of this study would inform the measures and strategies dedicated to improving the PCC practice and capacity building among the healthcare providers in Jeddah. By addressing identifying barriers and acting on factors reported, the healthcare authorities in Saudi Arabia could increase the care quality for women and children, reducing maternal and neonatal morbidity and mortality. There is a need for collaboration between different healthcare professionals and the establishment of a multidisciplinary approach that involves all workfare workers in all specialties.

## Appendices

**Table 5:** Association between perceptions and participants’ professions/jobs

Perception		What is your current position?					P value
		Family Physician (FM)	General Practitioner (GP)	Midwife	Other	Practitioner Nurse (PN)	
Important health issue	strongly disagree	3	4	0	3	10	0.026
	disagree	0	1	1	0	4	
	uncertain	1	2	1	0	4	
	agree	14	15	2	9	22	
	strongly agree	45	21	2	5	32	
Does not have any effect	strongly disagree	47	18	3	8	37	0.452
	disagree	6	8	0	1	18	
	uncertain	2	2	1	2	1	
	agree	6	6	2	3	9	
	strongly agree	2	9	0	3	7	
Luxury service]	strongly disagree	18	12	0	4	16	0.059
	disagree	21	7	2	1	14	
	uncertain	9	13	1	3	13	
	agree	10	8	2	4	10	
	strongly agree	5	3	1	5	19	
Hospital is the best place	strongly disagree	17	6	0	1	8	
	disagree	19	9	2	1	15	0.015
	uncertain	9	15	1	2	14	
	agree	14	9	2	8	20	
	strongly agree	4	4	1	5	15	
Planning for pregnancy often not happen	strongly disagree	7	4	0	0	2	0.333
	disagree	13	4	1	1	11	
	uncertain	20	17	2	9	25	
	agree	17	9	3	6	25	
	strongly agree	6	9	0	1	9	
High priority in my workload	strongly disagree	1	5	1	1	3	0.690
	disagree	13	6	1	2	17	
	uncertain	21	14	1	6	23	
	agree	20	12	3	7	18	
	strongly agree	8	6	0	1	11	
Not enough time	strongly disagree	6	5	1	0	11	
	disagree	13	10	1	3	10	0.057
	uncertain	8	5	1	0	23	
	agree	24	13	3	10	19	
	strongly agree	12	10	0	4	9	
No skills	strongly disagree	25	1	0	3	10	
	disagree	18	21	2	3	21	0.000
	uncertain	10	10	1	0	19	
	agree	10	5	3	10	16	
	strongly agree	0	6	0	1	6	
Little evidence supporting it	strongly disagree	18	1	0	3	8	0.000
	disagree	22	11	1	2	15	
	uncertain	13	23	2	4	11	
	agree	8	3	3	4	30	
	strongly agree	2	4	0	4	8	
I am not the most suitable to offer it	strongly disagree	25	7	0	2	8	
	disagree	20	19	2	1	17	0.000
	uncertain	7	7	1	10	20	
	agree	10	7	3	4	18	
	strongly agree	1	3	0	0	9	
General practice is the best place	strongly disagree	2	2	0	2	4	0.054
	disagree	2	5	0	1	11	
	uncertain	13	12	1	5	13	
	agree	25	21	5	8	26	
	strongly agree	21	3	0	1	18	
